# Biocompatibility of hydrogel derived from equine tendon extracellular matrix in horses subcutaneous tissue

**DOI:** 10.3389/fbioe.2023.1296743

**Published:** 2024-01-08

**Authors:** Thiago De Castilho, Gustavo dos Santos Rosa, Fernanda de Castro Stievani, Emanuel Vítor Pereira Apolônio, João Pedro Hübbe Pfeifer, Vittoria Guerra Altheman, Valéria Palialogo, Nilton José Dos Santos, Carlos Eduardo Fonseca-Alves, Ana Liz Garcia Alves

**Affiliations:** ^1^ Department of Veterinary Surgery and Animal Reproduction, Regenerative Medicine Lab, School of Veterinary Medicine and Animal Science, São Paulo State University (UNESP), Botucatu, Brazil; ^2^ Laboratory of Metabolic Disorders, School of Applied Sciences, University of Campinas (UNICAMP), São Paulo, Brazil; ^3^ Department of Veterinary Surgery and Animal Reproduction, School of Veterinary Medicine and Animal Science, São Paulo State University (UNESP), Botucatu, Brazil

**Keywords:** biomaterial, scaffold, collagen, regeneration, tenocyte

## Abstract

Tendinopathies account for a substantial proportion of musculoskeletal injuries. To improve treatment outcomes for partial and total tendon ruptures, new therapies are under investigation. These include the application of mesenchymal stem cells (MSCs) and biocompatible scaffolds derived from the Extracellular Matrix (ECM). Synthetic polymer hydrogels have not demonstrated results as promising as those achieved with ECM hydrogels sourced from the original tissue. This study aimed to evaluate the biocompatibility of a hydrogel formulated from equine tendon ECM. Six horses were administered three subcutaneous doses of the hydrogel, with a saline solution serving as a control. Biopsies were conducted on days 7, 14, and 56 post-application to gauge the hydrogel’s impact. Throughout the experiment, the horse’s physical condition remained stable. Thermographic analyses revealed a temperature increase in the treated groups compared to the control group within the initial 12 h. The von Frey test, used to measure the mechanical nociceptive threshold, also showed significant differences between the treated group and the control group at 6 h, 21 days, and 28 days. Histopathological analyses identified an inflammatory response on day 7, which was absent on days 14 and 56. Transmission electron microscopy indicated a decrease in inflammatory cellularity, while immunohistochemistry staining suggested an increased presence of inflammatory factors on day 14. In summary, the hydrogel is easily injectable, triggers a temporary local inflammatory response, and integrates into the adjacent tissue from day 14 onwards.

## 1 Introduction

Tendons are dense connective tissue structures that connect muscle to bone, thus transmitting force from muscle to bone and promoting locomotion. The composition of the tendon is represented by tenocytes interspersed in an extracellular matrix (ECM) composed of 65% water, 30% collagen, and 5% proteoglycans (Shojaee and Parham, 2019; Zachary, 2021). Type I collagen is predominant, representing approximately 95% of the total existing collagen. In the dehydrated tendon, collagen, and proteoglycans now represent 70%–80% of the tissue (Kümmerle et al., 2019; Yin et al., 2019).

Millions of musculoskeletal injuries are diagnosed annually, with a significant incidence of inflammatory processes and tendon or ligament ruptures, which are traumatic, partial, and/or total (Gaspar et al., 2015). The pathophysiology of tendinopathies is similar between humans and horses (Barreira et al., 2008; Godwin et al., 2012; Docheva et al., 2015; Ribitsch et al., 2020; Zhang et al., 2022). Equines are considered experimental models of the musculoskeletal system for human medicine, accepted by the scientific community and international organizations such as the US Food and Drug Administration (FDA) and the European Medicines Agency (EMA) (Shojaee and Parham, 2019). Tendinopathies can be caused by factors inherent to the individual, environment, activity, and intensity of the sport practiced (Shojaee and Parham, 2019). Depending on the severity of the injury, it can cause reduced performance and/or unfitness for the sport, leading to the animal’s retirement (Pluim et al., 2018). In horses, the forelimbs are the most affected, with a higher incidence in the superficial digital flexor tendon (SDFT) and deep digital flexor tendon (DDFT) (Barreira et al., 2008; Godwin et al., 2012; Gaspar et al., 2015). The tendon has a low capacity for regeneration, a slow repair process, and produces scar tissue, being functionally deficient compared to the previous one, with a recurrence rate between 42% and 56% (Dyson, 2004; Godwin et al., 2012; Kümmerle et al., 2019).

The treatment of tendinopathies aims to produce a repair tissue of similar quality and functionality to the original tissue, before the injury (Carvalho, 2012). Anti-inflammatories, platelet-rich plasma (PRP), whole blood, growth hormone, and mesenchymal stem cells (MSCs) are used as therapies for tendinopathies (Farnebo et al., 2014). The use of mesenchymal stem cell therapy as an adjunct to conventional treatments has shown favorable results in reducing the time taken to repair the injured tendon, improving tissue quality in the treatment of tendinopathies, and, as a result, reducing recurrences (Carvalho et al., 2011; Godwin et al., 2012; Guercio et al., 2015). Tendon healing can be accelerated by the use of biocompatible scaffolds in the injured region that will facilitate *in situ* regeneration by three-dimensional guided tissue regeneration. These cells, after their proliferation, remain in place on the scaffold forming healthy tissue. The extracellular matrix helps to support cell growth, tissue formation, and regeneration (Farnebo et al., 2014). The combined use of MSCs in biocompatible scaffolds or bioactive compounds provides cell protection, causes mechanical stimulation at the treatment site, and aids in the paracrine effects of MSCs (Santos et al., 2018). Currently, there are several scaffolds, which are synthetic, biological, or biosynthetic (Smith et al., 2016). The use of a complete ECM framework is indicated as the best option for reproducing the microenvironment of the injured tissue, directly interfering in the behavior, dynamics, and cell differentiation (Saldin et al., 2017).

The tendinous ECM is composed of functional and structural molecules secreted by resident cells and forms a three-dimensional structure with a unique biochemical composition for each tissue type, comprising collagen and non-collagenous elements, such as proteoglycans and glycoproteins. Collagen stands out as the predominant constituent of the tendon’s ECM, constituting 60%–85% of its dry weight (Zhang et al., 2021). The discovery of ECM hydrogel solubilization and formation has led to an expansion of its *in vivo* and *in vitro* applications (Lovati et al., 2016; Saldin et al., 2017). The process of tendon tissue decellularization presents a distinct opportunity to acquire a scaffold featuring a natural extracellular matrix structure. This scaffold is hypoimmunogenic and exhibits biomechanical properties closely resembling those of the original tissue. These attributes confer significant advantages for both potential clinical applications and research purposes. Various protocols for decellularizing tendon or ligament tissue have been explored, involving physical, chemical, and/or enzymatic methods. Common sources are menisci, patellar, superficial, and deep digital flexor tendons (Burk et al., 2014; Visser et al., 2015).

An *in vitro* study found that adding tendon ECM hydrogel enhances the tenogenic differentiation of MSCs derived from human adipocytes, compared to a hydrogel without ECM (Yang et al., 2013). The ECM hydrogel, derived from decellularized tissue, is viewed as a promising scaffold for promoting tissue regeneration. It is used to aid the construction and functional remodeling of injured regions (Farnebo et al., 2014; Saldin et al., 2017; Spang and Christman, 2018). The application of ECM hydrogel has been implemented in various organs, including the myocardium, liver, trachea, esophagus, and tendon. The implantation can be performed in numerous ways and combinations, such as a coating, populated with stem cells, growth factors, other biological or synthetic materials, and more recently, as bio-inks for 3D printing (Garvican et al., 2014; Spang and Christman, 2018).

After *in vivo* implantation of biomaterial made with allogeneic material, a local reaction was expected. Signs of clinical inflammation were observed, such as slight increases in volume and local temperature (Ring et al., 2008). The thermographic examination makes it possible to detect thermal variations in more superficial tissues, indicating inflammation resulting from the application of the hydrogel. As it is a non-invasive and very efficient method, thermography becomes ideal for this type of analysis (Ring et al., 2008; Basile, 2012). Microscopically, hydrogel applications, when generating acute or chronic inflammation, involve neutrophils, macrophages, and multinucleated giant cells (Anderson et al., 2008; Sheikh et al., 2015). From the changes generated by inflammation, there is migration of immune system cells such as monocytes and differentiation into macrophages in the tissue and neutrophils to the region of application of the biomaterial (Witherel et al., 2019). Immunohistochemistry helps the visualization of inflammatory processes, with the labeling of inflammatory cells and inflammatory mediators. This technique was performed to identify pro-inflammatory (TNF-alpha) and anti-inflammatory (IL-4) markers (Aggarwal et al., 2019). Th2 lymphocytes are responsible for a large part of IL-4 secretion, and they can also be produced by mast cells and CD4^+^ T lymphocytes, basophils, and eosinophils (Liu et al., 2016). IL-4 can act in different roles, and its function is directly linked to the cell that is secreting it. It is known that IL-4 plays a very important role in inducing the differentiation of M2 macrophages, which contributes to an anti-inflammatory process (Zhu et al., 2015; Ho and Miaw, 2016). M2 macrophages have been described as fundamental in the preservation and repair of brain tissue by having local anti-inflammatory actions, cleaning cellular debris, and providing growth factors (Zhu et al., 2015). TNF-alpha is a pro-inflammatory cytokine produced by macrophages/monocytes, widely implicated in the pathogenesis of inflammatory disorders (Hira and Sajeli Begum, 2021). M1 macrophages are activated by interferon-gamma, interleukin 1 beta, and LPS, which are responsible for causing a powerful inflammatory reaction capable of killing microorganisms and producing pro-inflammatory factors such as TNF-alpha (Zhu et al., 2015). TNF-alpha is critical for communication during host defense, inflammation, and organogenesis. Like other members of the TNF/TNFR superfamily, TNF-alpha is an intercellular communicating molecule involved in the construction of transient or enduring multicellular structures. The central role of TNF-alpha is to initiate the inflammatory reactions of the innate immune system. While TNF-alpha is a critical component of innate and adaptive immunity, this cytokine can cause chronic inflammation, and when high acute concentrations are generated, it can trigger septic shock (Ma, 2001; Balkwill, 2006). Tumor necrosis factor (TNFSL) superfamily ligands and receptors have distinct structural features that link them to cell growth, cell survival, or cell death. Some of these can activate inflammatory and apoptotic pathways, depending on target cell types and other extrinsic stimuli. Many of the TNF receptor superfamily molecules are expressed in cells of the immune system, which may be central to autoimmune and inflammatory diseases, as well as cancer.

This study aimed to examine the biocompatibility of a hydrogel, derived from the extracellular matrix of equine tendons, using an *in vivo* model. We developed a biomaterial for injection into the horse’s dermis and monitored heart rate, body temperature, and pain sensitivity for 56 days post-application. Dermis samples from the application site were collected on days 7, 14, and 56 post-application to assess adherence progression and biocompatibility.

## 2 Material and methods

### 2.1 Experimental design

Six healthy mixed-breed horses were used in this study, with a mean age of 12,6 years and mean weight of 354,2 kg. Health status was confirmed by physical and lab examination. They remained in sand paddocks measuring 15 × 15 m^2^, receiving feed consisting of pre-dried *ad libitum* and laminated feed once a day.

### 2.2 Preparation of hydrogel derived from equine tendon extracellular matrix (ET-ECM)

Equine superficial digital flexor tendons (SDFT) were obtained from animals euthanized for non-septic reasons that did not involve the musculoskeletal system. Immediately after euthanasia, 10 cm of the SDFT were surgically excised from the metacarpal/metatarsal region aseptically and immediately placed in 1% buffer saline solution (PBS) with antibiotic and antimycotic (PAA Laboratories, Waltham, Massachusetts - United States of America) at 1% for up to 12 h in the refrigerator. The SDFT were dissected to eliminate adjacent connective tissue and fragmented into 1 cm to be frozen at −80°C until the next stage.

Decellularization of tendon fragments was performed as described by Burk et al. (2014) (Burk et al., 2014). Initially, the fragments underwent five cycles of 2 min of freezing in liquid nitrogen and thawing for 10 min in PBS. After this process, the tendon fragments were placed in deionized water (Mili-Q) for 48 h and sequentially incubated for 48 h in Tris buffer (pH 7.6) with 1% Triton X-100 under constant agitation at 37°C. Finally, they were washed twice for 15 min in PBS and incubated in culture medium (DMEM 0.1% glucose, 10% fetal bovine serum (FBS), 1% penicillin-streptomycin and 0.1% gentamicin) for 12 h and then another 12 h in PBS at 37°C, and then they were stored at −80°C for lyophilization. Assessment of decellularization was performed by hematoxylin and eosin (H&E) staining performed on three tendon fragments after decellularization.

After decellularization, freeze-drying was carried out in a 100-h cycle (Christ Gamma 2–16 LSC) to sublimate the water content of the fragments without damaging the structure. Subsequently, the fragments were ground in a micro knife mill (Marconi MA 048) until the powder passed through the fine filter (0.5 mm), forming the decellularized extracellular matrix. It was then sterilized in ethylene oxide and stored at −20°C for later use.

Digestion of 2% ECM (20 mg/mL) was performed with 0.2% pepsin (SIGMA–P7000) in 0.02M HCl at a pH of 2.2 for 48 h. After this period, the pH of the solution was neutralized (7.4) by adding 0.2M NaOH (1/9 of the initially digested volume) and 10x PBS (1/10 of the final neutralized volume) in an ice bath (Farnebo et al., 2014). Gelation was confirmed by inverting the vial after 2 h at 37°C.

### 2.3 Subcutaneous hydrogel application procedure

**FIGURE 1:**
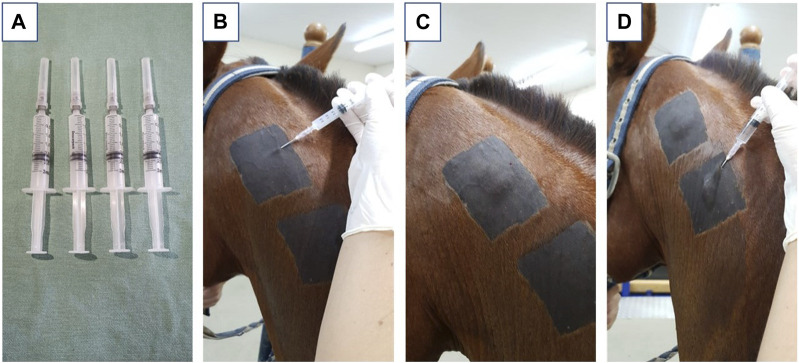
Representative images where **(A)** represents the syringes filled with 2 mL of hydrogel derived from equine tendon extracellular matrix; **(B)** represents the positioning of the needle in the correct application site, positioning in the subcutaneous region of the neck; **(C)** represents the aspect after application of the biomaterial; **(D)** represents the application of saline solution used as a control.

Each animal (n = 6) received three applications of hydrogel in the subcutaneous tissue of the neck region, each corresponding to a biopsy time point (7, 14, and 56 days post-application). In addition to the hydrogel, each animal was also administered a saline solution (NaCl 0.9%) in the subcutaneous tissue as a control. Both the hydrogel (HG) and control (CG) injections contained 2 mL of solution, administered using a 3 mL syringe and an 18G hypodermic needle. The injection site was shaved and cleaned before the procedure (Figure 1).

### 2.4 Analysis of the infrared thermography evaluation

Infrared thermographic evaluation (FLIR SC660 FlirSystems™) of the region submitted to the application was performed before application M0, 6, 12, and 24 h after application, then daily until completing 7 days (M48, M72, M96, M120, M144, M168) and then weekly until the end of the experiment (M14, M21, M28, M35, M42, M49, M56). Twenty-4 hours before the evaluation, the trichotomy of the region was redone to minimize the influence of the hairs on the image, and the animals were kept for at least 40 min in a stall for acclimatization before the examination. The camera was handheld and positioned one and a half meters from the animals, the distance from the device was always constant.

Two images were taken on each side of the neck and subsequently analyzed using the Flir tool software program. The chosen point was the center of the trichotomy of each point, defined as the application site. To remove the influence of ambient temperature and humidity, the “PadTemp” formula was used according to Basile et al. (2010) (Basile et al., 2010).

### 2.5 Assessment of the mechanical nociceptive threshold

The evaluation of the mechanical nociceptive threshold (MNL) was performed using a von Frey Electronic Esthesiometer with rigid tips and Supertips (Campden - Model I-2393) with a maximum pressure capacity of 800 g. The threshold was measured at three points in each evaluation site cranial, medium, and caudal, to the central point of injection of the biomaterial. The evaluation was performed at the same time as the thermographic examination. The average between the evaluation points was performed for the statistical analysis.

### 2.6 Assessment of heart rate, respiratory rate, and body temperature

A physical examination was conducted to detect any initial systemic adverse reactions to the biomaterial. This examination included monitoring the heart rate (HR), respiratory rate (RR), temperature, and capillary perfusion time (CPT). A digital thermometer was used to measure the temperature, while a stethoscope was employed to monitor the HR and RR. The CPT was manually assessed. All these parameters were documented and subsequently utilized for statistical analysis.

### 2.7 Biopsy collection in the hydrogel application region

Ultrasound localization was used to perform biopsies from the entire area at the injection site, including skin, subcutaneous tissue, and hydrogel remnants, at 7, 14, and 56 days post-hydrogel application. The horse was sedated with 10% Xylazine Hydrochloride (0.8 mg/kg) for the procedure. Following trichotomy, surgical antisepsis, and a local anesthetic block in an inverted “L" pattern using 2% Lidocaine Hydrochloride without a vasoconstrictor, the sample was collected. The sizes of the samples were 7 cm × 7 cm. It was then placed in flasks containing 50 mL of 10% formaldehyde.

### 2.8 Sample storage

The samples intended for histological and histopathological analyses were preserved in formalin for 24 h. Following this, they were rinsed in a solution composed of 70% ethanol. For the purposes of histological and immunohistochemical examination, the specimens were subsequently stored in 70% alcohol.

A small portion of each sample was placed in Karnovisky’s solution, fixing it for processing slides for transmission electron microscopy according to the protocol (Graham and Orenstein, 2007).

### 2.9 Histopathological analysis of the subcutaneous region containing hydrogel derived from equine tendon

Following the storage process outlined above, the samples were prepared for histological slide creation through a series of steps. Initially, the tissue was extracted from the alcohol, embedded in paraffin, and sectioned into 4 µm slides with a microtome. These slides were then placed in ovens to melt the paraffin and subsequently deparaffinized. The slides underwent a dehydration process involving three 5-min immersions in 100% alcohol, followed by 5-min immersions in 95%, 90%, 80%, and 70% alcohol baths. Afterward, the slides were rinsed in running water for 10 min, stained with Hematoxylin for 3 min, and then rinsed again in running water for 10 min. Post Hematoxylin staining, the slides were stained with Eosin for 30 s and rinsed in running water to eliminate any excess. Following staining, the slides were rehydrated through three 100% alcohol baths, a bath of alcohol combined with a Xylol solution, and three baths of PA xylene. Upon completion of this process, the slides were finalized with Permount. Each slide stained with H&E was photographed at 5-, 10-, 20-, and 40-times magnification and assessed for the presence of cell infiltrate, vascularity, and extravascular blood.

### 2.10 Immunohistochemical analysis

For immunohistochemistry analyses, fixed samples were embedded in paraffin, and 4 µm thick sections were produced on positively charged glass slides (StarFrost). The immunohistochemistry process began with the deparaffinization and hydration of these sections. Antigen retrieval was carried out in a pressure cooker at 95°C for 30 min or through three 5-min cycles in microwaves, using citrate buffer pH 6.0 or EDTA buffer. Following antigen retrieval, the slides were rinsed with PBS (0.1M, pH 7.4) and then incubated with a blocking solution (5% BSA in PBS) for 1 h. Post-blocking, the sections were incubated with anti-TNF- (TNF-R2 (L-20): sc-1074, Santa Cruz Biotechnology, Inc. Dallas, Texas 75,220 United States of America) and anti-IL4 (IL-4 (C-19): sc-1260, Santa Cruz Biotechnology, Inc. Dallas, Texas 75,220 United States of America), diluted in 1% BSA at a 1:100 ratio, overnight at 4°C. After incubation, the sections were rinsed with PBS and incubated with a peroxidase-conjugated secondary antibody diluted in the same buffer for 2 h at room temperature. The samples were then rinsed with PBS again, and the markings were revealed with diaminobenzidine (DAB). The samples were counterstained with hematoxylin to mark the nucleus and enhance visualization under a light microscope. Following development, the slides underwent assembly and were finalized. For the negative control, the primary antibody was replaced with the immunoglobulin of the species from which the primary antibody was derived, at a 1:100 dilution. Slides were analyzed using a Leica DM2500 microscope and images were captured using a Leica DMC2900 camera and Leica Qwin image analysis software version 3.1. The blinded review was performed by omitting from the observers the slide group (control vs. treated) and the animal numbers. The IHC evaluation was performed by a semi-quantitative analysis, conducted by two independent observers (Grisoni Sanchez et al., 2023).

### 2.11 Characterization of the hydrogel *in vivo* by transmission electron microscopy analysis

During the experiment, samples were collected and sectioned into fragments no larger than 2 mm. These fragments were then fixed in Karnovsky solution for a minimum of 3 hours at room temperature. Following fixation, the samples were rinsed in a 0.1M phosphate buffer with a pH of 7.3. The material was then submerged in osmium tetroxide. After 2 hours, the material was rinsed with distilled water and immersed in 0.5% uranyl acetate. The samples were subsequently dehydrated using a graded series of acetone. Upon completion of dehydration, the material was placed in a 1:1 mixture of Araldite^®^ resin and 100% acetone. The samples were then embedded in pure resin. The area of interest was selected, and the blocks were trimmed to minimize the surface area, enabling the creation of ultrathin sections (90 nm). The sections were then contrasted using a saturated solution of uranyl acetate in 50% alcohol, followed by lead citrate. Finally, the prepared slides were examined under a transmission electron microscope.

### 2.12 Statistical analysis

Statistical analyses and graphing were conducted using the GraphPad Software program (Graph Prism 8.0.1, Boston, MA 02110, United States of America). The Shapiro-Wilk test was employed to assess data for normality, followed by a one-way ANOVA or Kruskal–Wallis test for group comparisons. The Bonferroni test or Dunn’s multiple comparisons test was utilized for moment evaluations. Significance was assigned when *p* < 0.05.

## 3 Results

### 3.1 Thermography

**FIGURE 2:**
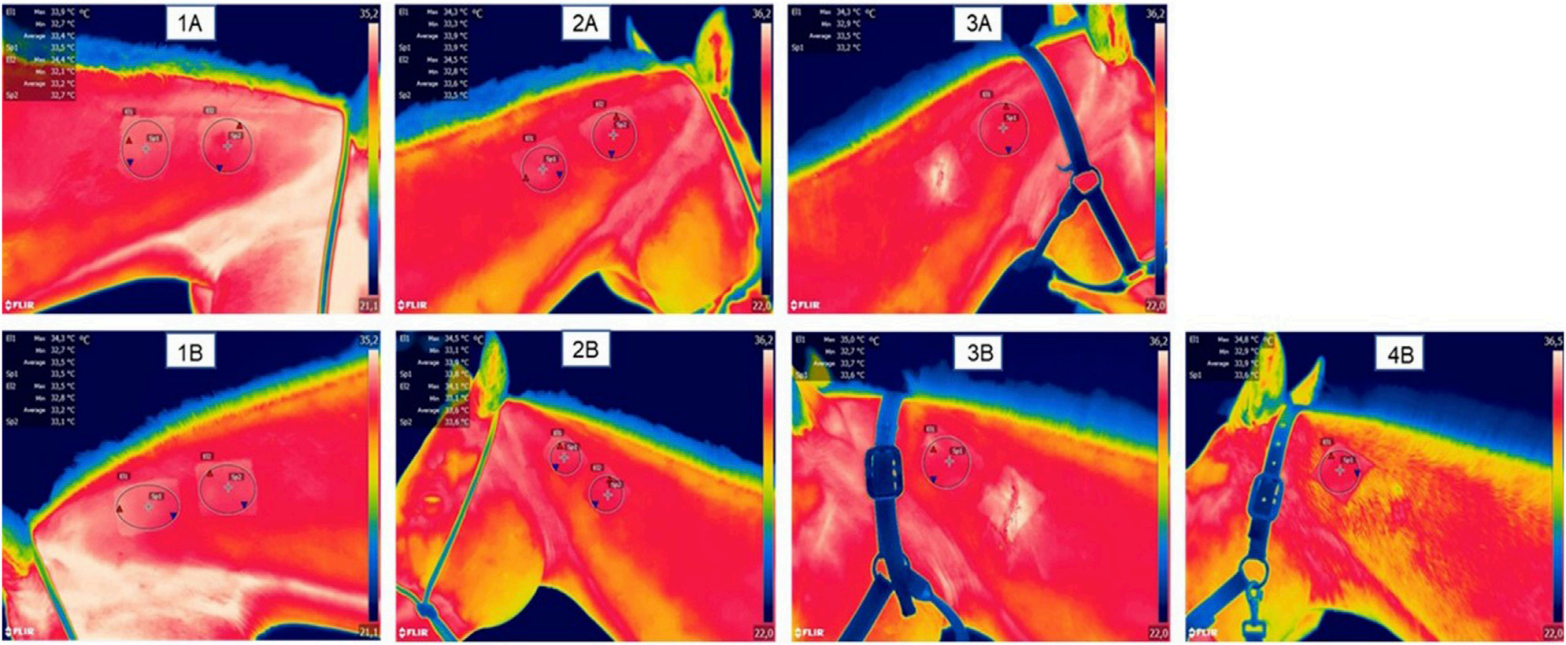
Representative images of thermographic monitoring; **(1A)** represents the right side of the neck and **(1B)** represents the left side of the neck at 0 h, pre-application of the biomaterial; **(2A)** represents the right side with two hydrogel applications at the time 7 days post application; **(2B)** represents the left side with a hydrogel application and a saline application as a control; **(3A)** represents the right side after the first biopsy (done at time 7 days) with a remaining application of hydrogel at time 14 days; **(3B)** represents the left side after control biopsy (done at day 7) and with a remaining application of hydrogel at day 14.

**FIGURE 3:**
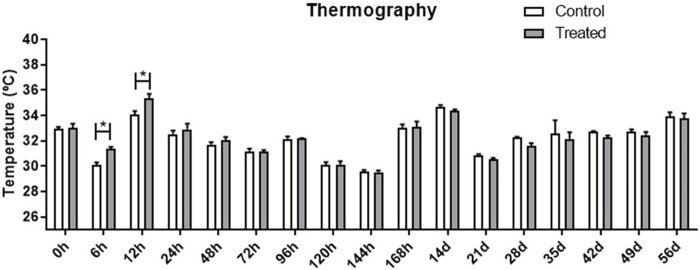
Evaluation of the temperature at the application site, in the treated and control groups at different evaluation times. Data is represented by the mean ± standard error of the mean; “*” represents statistical difference when *p* < 0.05.

Figure 2 presents a thermographic evaluation of the hydrogel insertion site at various time intervals, utilizing the FLIR SC660 (FlirSystems™). A temperature increase was observed at the hydrogel application site in the treated group, specifically at 6 and 12 h post-application (31.33°C ± 0.17; 35.31°C ± 0.38), compared to the control group (30.10°C ± 0.18; 34.05°C ± 0.28) (*p* < 0.05) (Figure 3). This indicates a reaction to the biomaterial during this period.

### 3.2 Assessment of the mechanical nociceptive threshold

**FIGURE 4:**
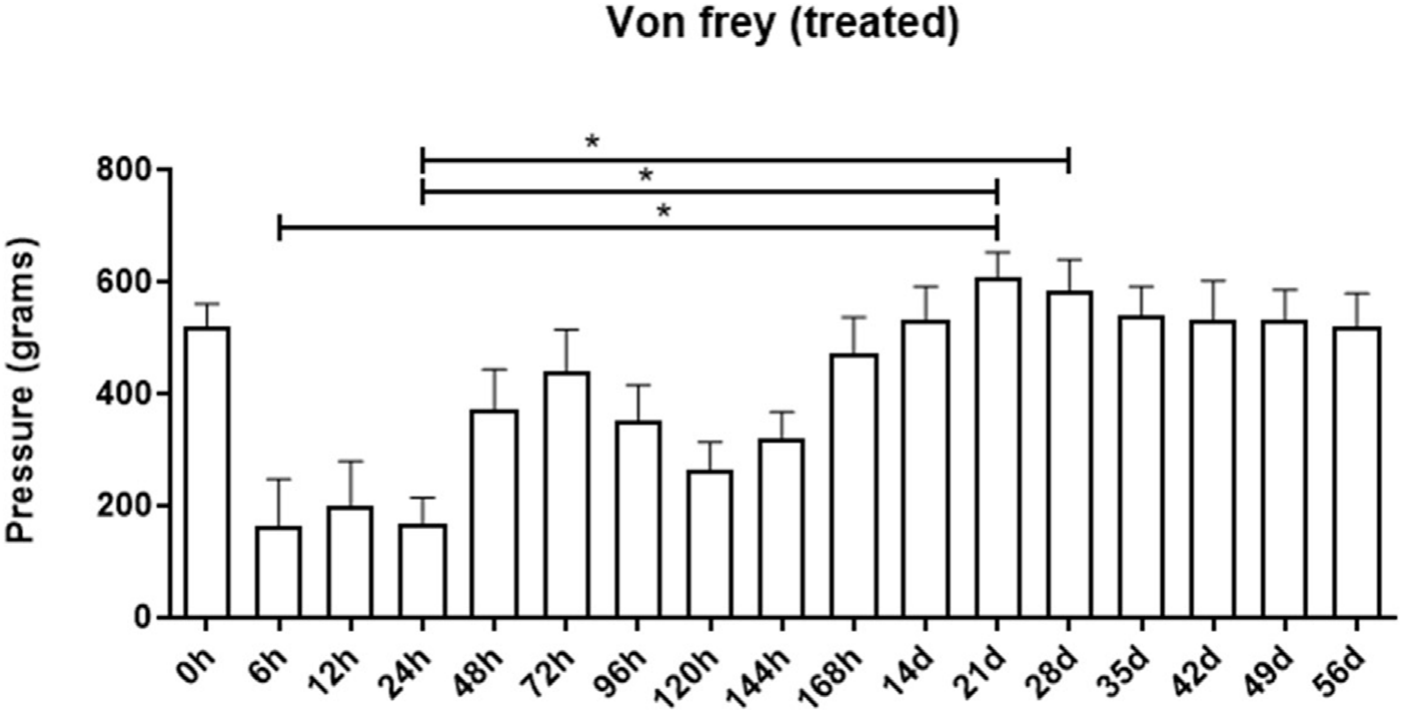
Assessment of the mechanical nociceptive threshold of the treated group at different assessment times. Data is represented by the mean ± standard error of the mean; “*” represents statistical difference when *p* < 0.05.

The electronic Von Frey method, used for mechanical nociceptive evaluation, facilitates the measurement of local pressure sensitivity up to a maximum of 800 g. Upon analyzing the results derived from the Von Frey electronic method, no discernible increase in local sensitivity was noted between the control and treated groups (data not shown). Despite an apparent increase in local sensitivity in the group treated at 6, 12, and 24-h intervals, these increases were not statistically significant when compared to the baseline. A decrease in sensitivity (indicating greater pressure tolerance) was observed 21 and 28 days post-hydrogel application, with values of 606.9 g ± 45.3 and 584.1 g ± 55.4, respectively. These values contrast with the initial measurements taken at 6h and 24 h post-application (164.3 g ± 82.8 and 168.9 g ± 45.4, respectively), which indicated lower pressure tolerance (*p* < 0.05). This suggests that, within the first 24 h, the sites where the biomaterial was applied exhibited increased sensitivity (Figure 4).

### 3.3 Physical exam

Throughout the experiment, the animals exhibited variations in heart rate, respiratory rate, and temperature. However, these remained within the reference values deemed normal for the equine species. These fluctuations were associated with environmental and management factors, rather than the implantation of the biomaterial.

### 3.4 Histopathological analysis

**FIGURE 5:**
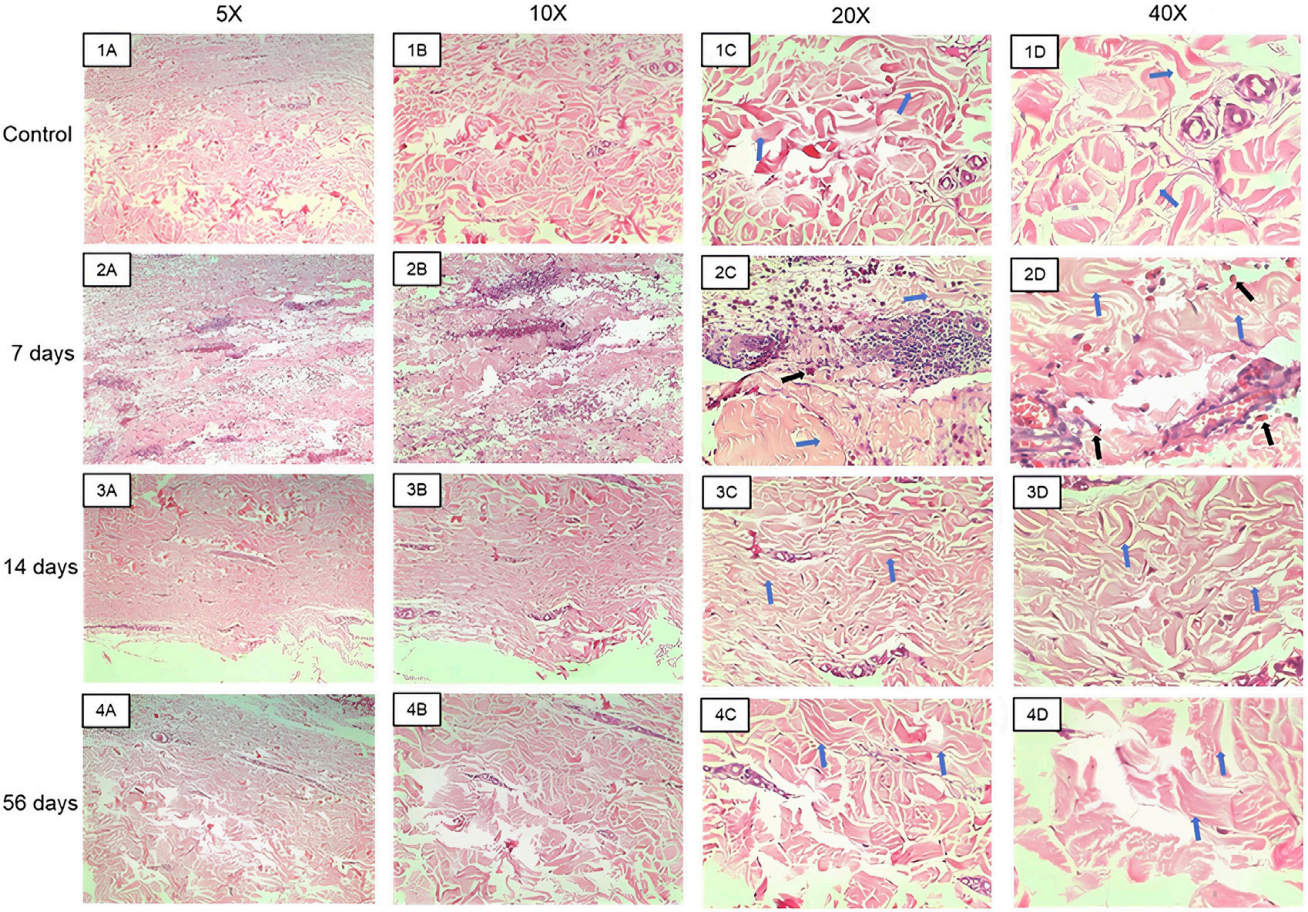
Representative images of the hematoxylin and eosin slides made with the material collected in the biopsies. Images **(1A–D)** represent control samples at 5x, 10x, ×20, and ×40 magnifications. Images **(2A–D)** represent samples from 7 days at 5x, 10x, ×20, and ×40 magnifications. Images **(3A–D)** represent samples from the 14th day at 5x, 10x, ×20, and ×40 magnifications. Images **(4A–D)** represent samples from the 56th day at 5x, 10x, ×20, and ×40 magnifications. Blue arrows indicate collagen fibers and black arrows indicate macrophages phagocytosing red blood cells.

Figure 5 presents the histopathological analysis of skin and subcutaneous tissues in both control samples and those treated with the biomaterial after 7, 14, and 56 days. The slides were stained with Hematoxylin and Eosin (H&E), enabling the observation of various lesions and inflammatory infiltrates within the dermis of the examined samples. The connective tissue exhibited moderate to severe hemorrhage, cell infiltration, phagocytosis of red blood cells, and the presence of lymphocytes. These lesions became apparent after 7 days of biomaterial application (Figure 5.2A-D). However, after 14 days, no hemorrhage or immune system cell infiltration exceeding the expected level for the tissue was observed (Figure 5.3A-D). It was noted that 14 days post-biomaterial application, the tissue displayed regenerated regions exhibiting normal dermis characteristics. These included evenly arranged, unbroken blood vessels, active fibroblastic cells (with decondensed nuclei and high activity), and collagen fibers organized according to standard skin histology.

### 3.5 Immunohistochemistry analysis

Immunohistochemical analyses were performed on skin samples at the site where the hydrogel was applied. Samples from animals at 7, 14, and 56 days after application of the biomaterial were submitted to Immunohistochemistry for inflammation markers for IL-4 and TNF-alpha. Little positive reactivity was observed for IL-4 and TNF-alpha immunostaining in the samples of the group 7 days after application of the biomaterial; the control samples did not show staining.

**FIGURE 6:**
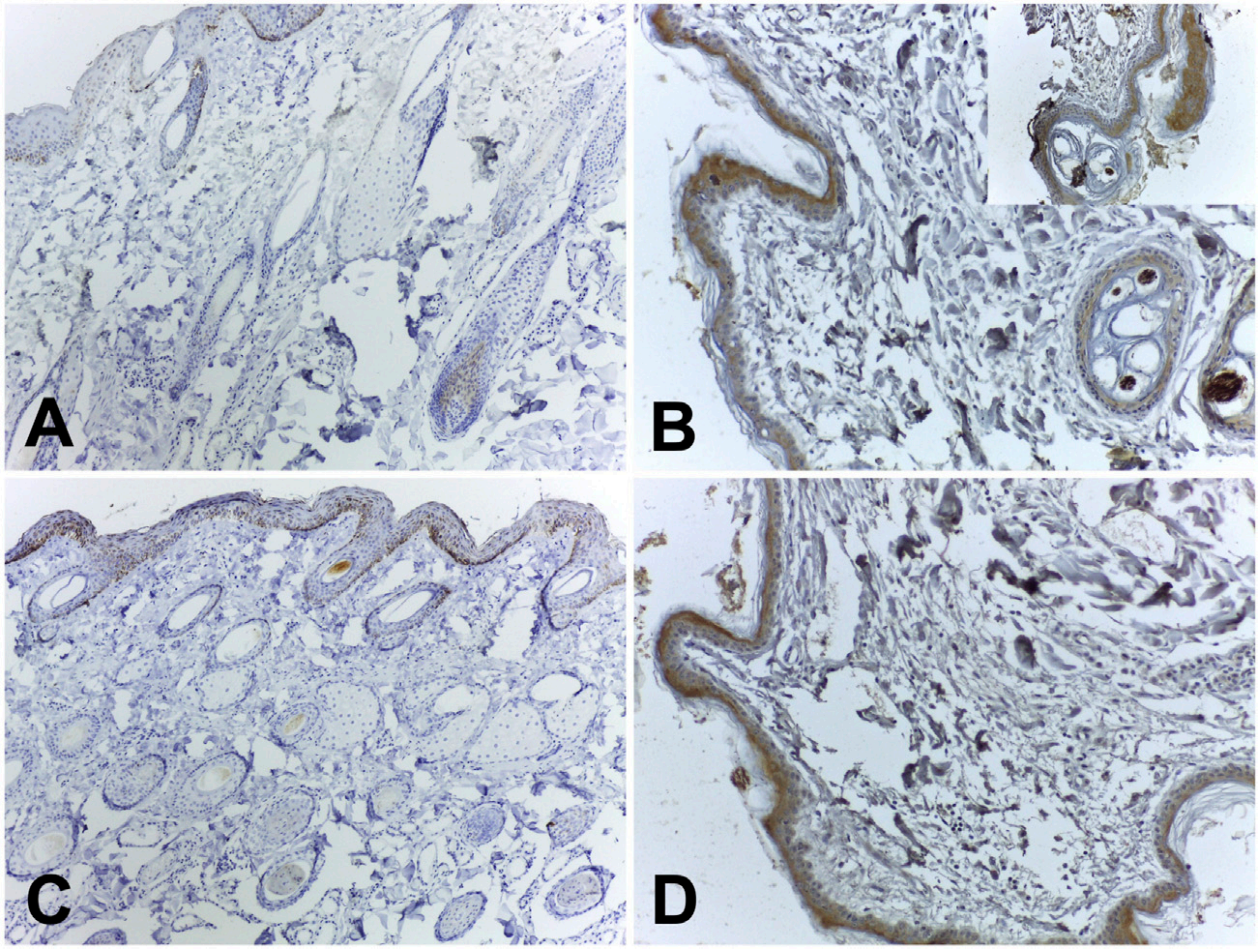
Representative immunohistochemical images of TNF-alpha and IL-4 expression. **(A)** TNF-alpha: score 1+; **(B)** TNF-alpha: score 3+ (insert from a well-marked area); **(C)** IL-4: score 1+; **(D)** IL-4 score 2+.

On day 14, both the biomaterial samples and the control group tested positive for TNF-alpha immunostaining. However, only the biomaterial samples showed positive immunohistochemical reactions for IL-4; no labeling was detected in the control group. By day 56, the group treated with the biomaterial exhibited increased positive immunostaining for both IL-4 and TNF-alpha (Table 1). Representative images of TNF-alpha (Images 6A-B) and IL-4 (Images 6C-D) expression were represented in Figure 6.

**TABLE 1:** Immunohistochemistry results.

Animals	Time (days)	Grups	IL-4	TNF-alfa
1	7	Control	Negative	Negative
2		Control	Negative	Negative
1		Treated	Negative	Negative
2		Treated	1+	Negative
3		Treated	Negative	Negative
4		Treated	Negative	1+
5		Treated	Negative	1+
6		Treated	Negative	Negative
4	14	Control	Negative	2+
5		Control	Negative	1+
1		Treated	1+	1+
2		Treated	Negative	1+
3		Treated	1+	1+
4		Treated	2+	2+
5		Treated	Negative	2+
6		Treated	1+	Negative
3	56	Control	Negative	1+
6		Control	Negative	2+
1		Treated	1+	2+
2		Treated	1+	2+
3		Treated	1+	Negative
4		Treated	2+	3+
5		Treated	1+	2+
6		Treated	1+	2+

### 3.6 Transmission electron microscopy analysis

**FIGURE 7:**
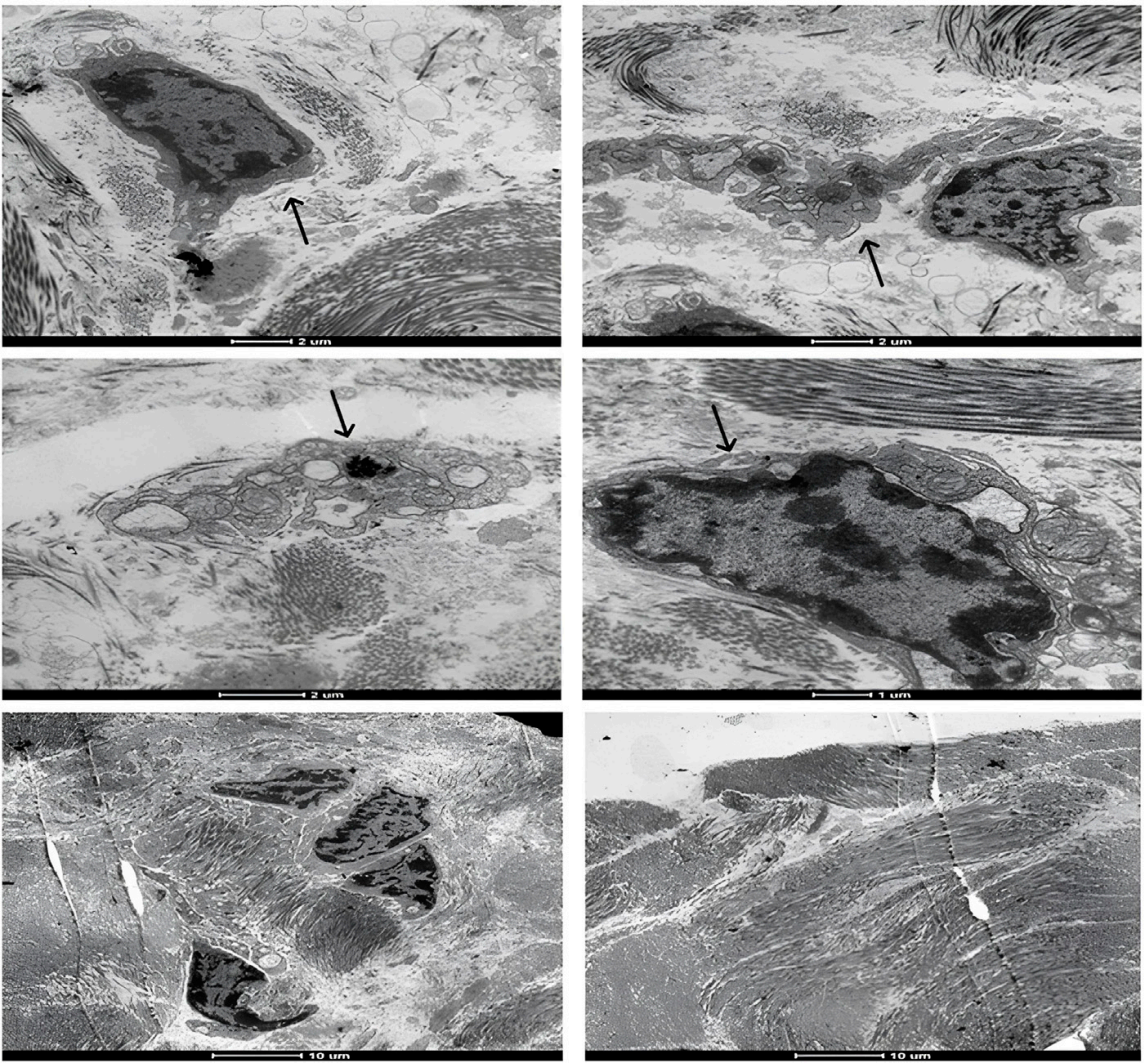
Representative transmission electron microscopy images of the moment 7 days after application of the biomaterial; Black arrows indicate inflammatory cells.

**FIGURE 8:**
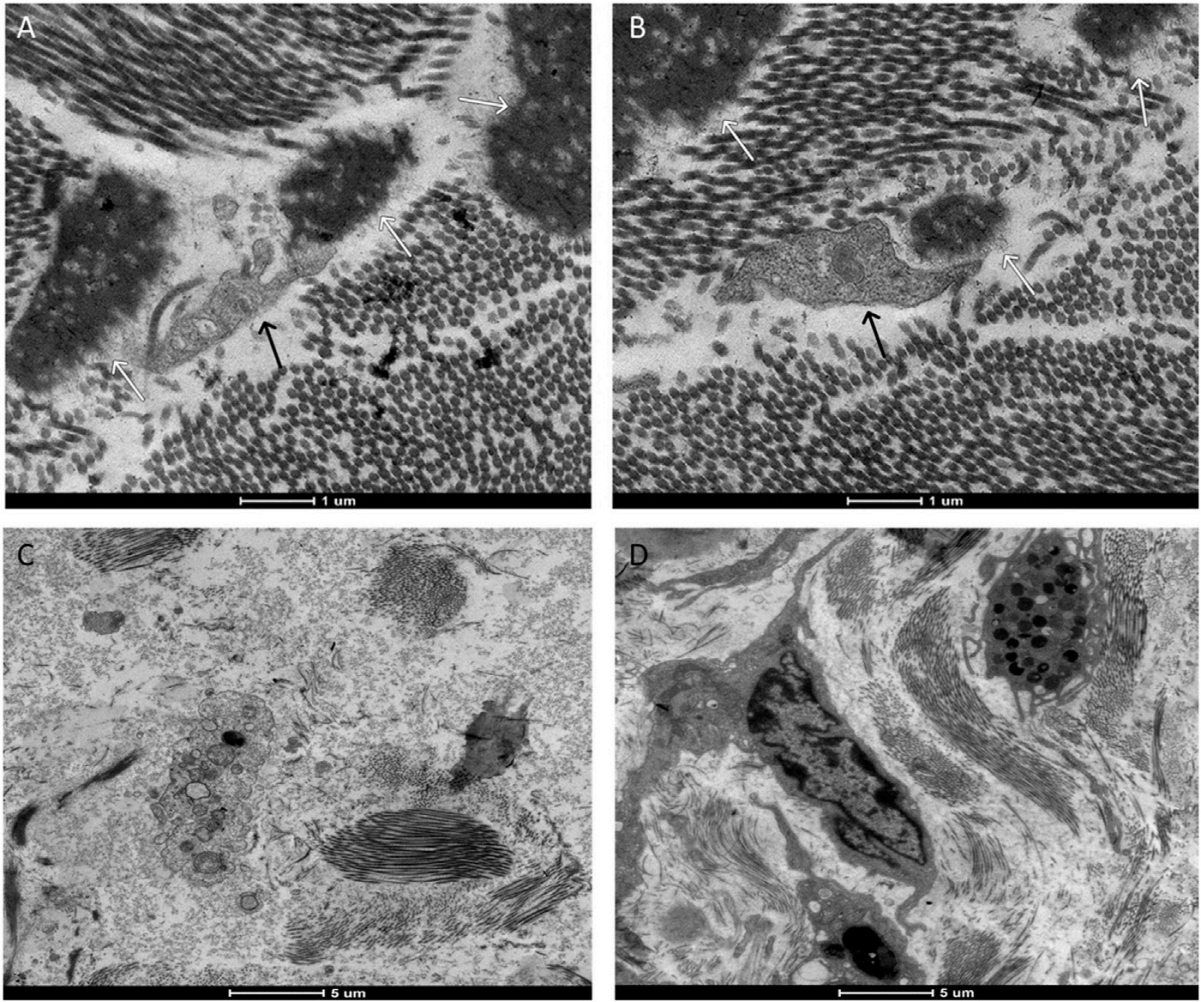
Representative transmission electron microscopy images. Images **(A,B)** represent the moment 14 days after the application of the biomaterial; Images **(C,D)** represent the control group. Cell phagocytosing (black arrow) the biomaterial (white arrow).

Upon examination of the samples under a transmission electron microscope in M7 (Figure 7), numerous dendritic cells surrounded by biomaterial were identified. In M14, cells phagocytosing fragments of the biomaterial were observed (Figure 8), albeit in significantly fewer numbers than previously noted. At the subsequent 56 days, despite the continued presence of dendritic cells, the biomaterial was no longer detectable adjacent to the tissue. These findings are consistent with the images reported on hematoxylin and eosin slides.

## 4 Discussion

In this study, the compatibility of a hydrogel derived from an ECM of an equine tendon inserted in the subcutaneous region of horses was verified. We verified that there was an acute inflammation and sensitivity at the site, but there was no rejection of the biomaterial after 56 days of the experiment.

The implantation of a decellularized extracellular matrix hydrogel is a promising biomaterial for *in vivo* use (Farnebo et al., 2014). It is common to observe a local temperature increase after hydrogel application. The increase in surface temperature at the application site represents a defense mechanism response, characterized by a local inflammatory process, with changes in blood flow, increased permeability of blood vessels, and migration of fluids, proteins, and defense cells from the circulation to the site of tissue injury (Anderson et al., 2008; Basile et al., 2010; Browne and Pandit, 2015). Measured by thermography, the increase in temperature was one of the indicators of this inflammatory process.

In addition to changes in temperature, other parameters were used to detect other signs of inflammation, such as changes in local sensitivity, on palpation and increase in volume on inspection, resulting from the inflammatory process. The measurement of local sensitivity was performed in various ways, from palpation to the use of electronic pressure algometry. However, to minimize the subjectivity of the evaluation, the use of electronic pressure algometry was the most indicated, thus being able to identify, with a more objective parameter, the nociceptive thresholds of local sensitivity, per unit in grams (Visser et al., 2019).

In the present study carried out with the use of electronic pressure algometry, the mechanical nociceptive threshold was lower in the first hours after application (6 and 12 h), with greater resistance of the animals to allow the application of pressure through the rigid tip, which coincided with the acute local reaction. This was also characterized by edema and increased skin temperature. It is important to remember that the evaluation using algometry needs to take into account several factors, such as tissue thickness and character, anatomical location, as well as areas with less tissue thickness, notably regions with less muscle (Pongratz and Licka, 2017). Therefore, all measurements were evaluated by a qualified professional to maintain uniformity in the results.

The mechanical nociceptive threshold assessment was complemented with other parameters such as changes in skin temperature, muscle hypertonicity, and imaging tests, which increased the accuracy of the test (Haussler, 2020). The experiment carried out included physical analyses to monitor changes related to reactions to the biomaterial. The increase in body temperature at 12 h indicates the presence of inflammation resulting from the implantation of the biomaterial (Bundgaard et al., 2018). The HR, FR, and TPC parameters remained within the physiologically normal range of the species. The reaction to the hydrogel was considered moderate but transient.

The innate immune system functions to identify potential threats to an organism and break them down into smaller particles for elimination. However, this mechanism is not highly effective against foreign bodies. The foreign body reaction comprises five stages: protein adsorption, acute inflammation, chronic inflammation, giant cell formation, and fibrous capsule formation (Klopfleisch and Jung, 2017). Histopathological analysis revealed signs of acute inflammation, including hemorrhage and the migration of neutrophils, polymorphonuclear cells, and macrophages. However, no signs of chronic inflammation, such as the formation of a fibrous capsule that isolates the biomaterial from the host tissue, were observed. This reaction to biomaterials is undesirable (Klopfleisch and Jung, 2017).

The acute inflammation that occurred is in line with basic literature data, a rapid response to a noxious agent that carries host defense mediators, increasing local blood flow, and allowing plasma proteins and leukocytes to leave the circulation (Kumar et al., 2014). This cell migration was observed in previous studies (Carvalho et al., 2020). In our study, it was possible to identify inflammatory cells and hemorrhage 7 days after implantation. No fibrous capsule formation was observed, which could lead to rejection of the implant by the host. In the following moments, 14 and 56 days after application, the presence of inflammatory cells was not observed at the same intensity. There was the presence of neovascularization at the site, a fact that was also reported in previous studies (Carvalho et al., 2020).

Biomaterials are deemed biocompatible if both acute and chronic inflammatory reactions cease within 2 weeks. Prolonged acute inflammatory processes, persisting beyond this timeframe, may suggest the presence of chronic inflammation or potential infections (Sheikh et al., 2015).

Immunostaining for IL-4 and TNF-alpha was detected in cells adjacent to the basement membrane, keratinocytes, and hair follicles. Employing TNF-alpha and IL-4 in this technique, as opposed to specific antibodies for the macrophage cell membrane, could potentially result in cross-reactions with other cells that produce these substances and are affected by their actions, as previously noted (Zhu et al., 2015; Ho and Miaw, 2016; Liu et al., 2016). Future analyses can be carried out to verify the expression of the cytokine IL-1 beta, a potent pro-inflammatory cytokine that is crucial for host-defense responses to infection and injury (Van den eeckhout et al., 2021).

Upon comparing the results of Immunohistochemistry with those of hematoxylin and eosin slides, a disparity was observed. The markers for IL-4 and TNF-alpha were more prevalent on the 56th day, whereas inflammatory cells were scarce in the histological sections at the same time point. This discrepancy can be attributed to the potential for cross-reactions of the markers with other cells (Balkwill, 2006; Ho and Miaw, 2016; Liu et al., 2016).

Transmission electron microscopy (TEM) serves as a crucial tool in evaluating the interactions between cellular ultrastructures and various materials, including hydrogels, implant materials, biosensor devices, nanopiles, nanoneedles, and electrical devices (Hansel et al., 2020). Previous research has utilized TEM to scrutinize the integrity of the epidermis and the dermis-epidermis junction, offering detailed insights into the skin’s ultrastructure (Loh et al., 2018). TEM-generated images have provided valuable information regarding the integrity of biomaterials applied to animals over periods of 7, 14, and 56 days, and have also revealed the interaction of immune system cells with the hydrogel. These findings offer a more comprehensive understanding of the histological observations, where a heightened cellular defense and hydrogel presence were noted at the 7-day mark. At the subsequent 14-day interval, inflammatory cells were still evident, albeit with a significantly reduced number of hydrogel fragments. This trend continued until the final 56-day mark, at which point neither the biomaterial nor the previously observed cellularity could be identified.

In conclusion, the host response to the allogeneic biomaterial utilized in this study was deemed satisfactory, with no additional complications observed. While the occurrence of an acute local reaction to the extracellular matrix hydrogel application remains a challenge, it was deemed insignificant due to its disappearance within the 14-day evaluation period and the successful integration of the hydrogel into the recipient tissue.

The potential benefits of utilizing the tendon extracellular matrix as a carrier for substances and stem cells warrant evaluation to determine its prospective therapeutic applications.

## 5 Conclusion

The implantation of the allogeneic biomaterial resulted in an initial inflammatory response. However, the hydrogel successfully integrated into the adjacent tissue without any residual inflammation, thereby confirming its biocompatibility and bioabsorbility. This suggests its viability as a scaffold for mesenchymal stem cells in the treatment of equine tendon injuries.

## Data Availability

The raw data supporting the conclusion of this article will be made available by the authors, without undue reservation.

## References

[B1] Aggarwal R. Jain A. K. Mittal P. Kohli M. Jawanjal P. Rath G. (2019). Association of pro- and anti-inflammatory cytokines in preeclampsia. J. Clin. Lab. Anal. 33 (4), e22834. Epub 2019 Jan 21. PMID: 30666720; PMCID: PMC6528584. 10.1002/jcla.22834 30666720 PMC6528584

[B2] Anderson J. M. Rodriguez A. Chang D. T. (2008). Foreign body reaction to biomaterials. Semininars Immunol. 20 (2), 86–100. 10.1016/j.smim.2007.11.004 PMC2327202 18162407

[B3] Balkwill F. (2006). TNF-alpha in promotion and progression of cancer. Cancer Metastasis Rev. 25 (3), 409–416. 10.1007/s10555-006-9005-3 16951987

[B4] Barreira A. P. B. Alves A. L. G. Salto M. E. Arnorint R. L. Kohayagawa A. Menarim B. C. (2008). Autologous implant of bone marrow mononuclear cells as treatment of induced equine tendinitis. Int. J. Appl. Res. Veterinary Med., 6 46–54.

[B5] Basile C. R. (2012). “Metodologia de avaliação e análise de termografia em equinos. 2012. xii, 102 f,” in Trabalho de conclusão de curso (bacharelado - medicina Veterinária) - universidade Estadual Paulista, Faculdade de Ciências Agrárias e Veterinárias. São Paolo, Brazil Universidade Estadual Paulista Disponível em: <http://hdl.handle.net/11449/sl118231>.

[B6] Basile R. C. Basile M. T. Ferraz G. C. Pereira M. C. Queiroz-Neto A. (2010). Equine inflammatory process evaluation using quantitative thermografic methodology. Ars Veterinaria, Jaboticabal, Sp. 26 (n.2), 077–081. 10.15361/2175-0106.2010v26n2p077-081

[B7] Browne S. Pandit A. (2015). Biomaterial-mediated modification of the local inflammatory environment. Front. Bioeng. Biotechnol. 3, 67. PMID: 26029692; PMCID: PMC4432793. 10.3389/fbioe.2015.00067 26029692 PMC4432793

[B8] Bundgaard L. Sørensen M. A. Nilsson T. Salling E. Jacobsen S. (2018). Evaluation of systemic and local inflammatory parameters and manifestations of pain in an equine experimental wound model. J. Equine Vet. Sci. 68, 81–87. Epub 2018 Jun 4. PMID: 31256894. 10.1016/j.jevs.2018.05.219 31256894

[B9] Burk J. Erbe I. Berner D. Kacza J. Kasper C. Pfeiffer B. (2014). Freeze-thaw cycles enhance decellularization of large tendons. Tissue Eng. Part C. Methods 20 (4), 276–284. 10.1089/ten.tec.2012.0760 23879725 PMC3968887

[B10] Carvalho A. M. (2012). “Implante de células tronco mesenquimais autólogas, associadas ao plasma rico em plaquetas em tendinites experimentais de equinos. 2012. 89f,” in Tese de Doutorado. Tese (Doutorado), Faculdade de Medicina Veterinária e Zootecnia (Botucatu, Brazil: Universidade Estadual Paulista), 9.

[B11] Carvalho A. M. Alves A. L. G. de Oliveira P. G. G. Cisneros Álvarez L. E. Amorim R. L. Hussni C. A. (2011). Use of adipose tissue-derived mesenchymal stem cells for experimental tendinitis therapy in equines. J. Equine Veterinary Sci. 31 (1), 26–34. 10.1016/j.jevs.2010.11.014

[B12] Carvalho J. R. G. Conde G. Antonioli M. L. Dias P. P. Vasconcelos R. O. Taboga S. R. (2020). Biocompatibility and biodegradation of poly(lactic acid) (PLA) and an immiscible PLA/poly(ε-caprolactone) (PCL) blend compatibilized by poly(ε-caprolactone-b-tetrahydrofuran) implanted in horses. Polym. J. 52, 629–643. 10.1038/s41428-020-0308-y

[B13] Docheva D. Müller S. A. Majewski M. Evans C. H. (2015). Biologics for tendon repair. Adv. drug Deliv. Rev. 84, 222–239. 10.1016/j.addr.2014.11.015 25446135 PMC4519231

[B14] Dyson S. J. (2004). Medical management of superficial digital flexor tendonitis: a comparative study in 219 horses (1992‐2000). Equine veterinary J. 36 (5), 415–419. 10.2746/0425164044868422 15253082

[B15] Farnebo S. Woon C. Y. Schmitt T. Joubert L. M. Kim M. Pham H. (2014). Design and characterization of an injectable tendon hydrogel: a novel scaffold for guided tissue regeneration in the musculoskeletal system. Tissue Eng. Part A 20 (9-10), 1550–1561. 10.1089/ten.tea.2013.0207 24341855

[B16] Garvican E. R. Dudhia J. Alves A. L. Clements L. E. Plessis F. D. Smith R. K. (2014). Mesenchymal stem cells modulate release of matrix proteins from tendon surfaces *in vitro*: a potential beneficial therapeutic effect. Regen. Med. 9 (3), 295–308. 10.2217/rme.14.7 24935042

[B17] Gaspar D. Spanoudes K. Holladay C. Pandit A. Zeugolis D. (2015). Progress in cellbased therapies for tendon repair. Adv. Drug Deliv. Rev. 84, 240–256. 10.1016/j.addr.2014.11.023 25543005

[B18] Godwin E. E. Young N. J. Dudhia J. Beamish I. C. Smith R. K. W. (2012). Implantation of bone marrow‐derived mesenchymal stem cells demonstrates improved outcome in horses with overstrain injury of the superficial digital flexor tendon. Equine veterinary J. 44 (1), 25–32. 10.1111/j.2042-3306.2011.00363.x 21615465

[B19] Graham L. Orenstein J. (2007). Processing tissue and cells for transmission electron microscopy in diagnostic pathology and research. Nat. Protoc. 2, 2439–2450. 10.1038/nprot.2007.304 17947985 PMC7086545

[B20] Grisoni Sanchez C. Figueiredo M. L. de Sartori Camargo L. Benevenuto L. G. D. Lacerda Z. A. Fonseca-Alves C. E. (2023). Is osteopontin a good marker for bone metastasis in canine mammary gland tumor and prostate cancer? Anim. (Basel) 13 (20), 3211. PMID: 37893935; PMCID: PMC10603680. 10.3390/ani13203211 PMC10603680 37893935

[B21] Guercio A. Di Marco P. Casella S. Russotto L. Puglisi F. Majolino C. (2015). Mesenchymal stem cells derived from subcutaneous fat and plateletrich plasma used in athletic horses with lameness of the superficial digital flexor tendon. J. Equine Veterinary Sci. 35 (1), 19–26. 10.1016/j.jevs.2014.10.006

[B22] Hansel C. S. Holme M. N. Gopal S. Stevens M. M. (2020). Advances in high-resolution microscopy for the study of intracellular interactions with biomaterials. Biomaterials 226, 119406. 119406. Epub 2019 Aug 6. PMID: 31558349. 10.1016/j.biomaterials.2019.119406 31558349

[B23] Haussler K. K. (2020). Pressure algometry for the detection of mechanical nociceptive thresholds in horses. Anim. (Basel) 10 (12), 2195. PMID: 33255216; PMCID: PMC7760268. 10.3390/ani10122195 PMC7760268 33255216

[B24] Hira K. Sajeli Begum A. (2021). Methods for evaluation of TNF-α inhibition effect. Methods Mol. Biol. 2248, 271–279. PMID: 33185884. 10.1007/978-1-0716-1130-2_21 33185884

[B25] Ho I. C. Miaw S. C. (2016). Regulation of IL-4 expression in immunity and diseases. Adv. Exp. Med. Biol. 941, 31–77. PMID: 27734408. 10.1007/978-94-024-0921-5_3 27734408

[B26] Klopfleisch R. Jung F. (2017). The pathology of the foreign body reaction against biomaterials. J. Biomed. Mater. Res. Part A. 105 (3), 927–940. Epub 2016 Nov 25. PMID: 27813288. 10.1002/jbm.a.35958 27813288

[B27] Kumar V. Abbas A. K. Fausto N. Aster J. C. (2014). Robbins and Cotran. Pathologic basis of disease. professional edition e-book. Amsterdam, Netherlands Elsevier health sciences.

[B28] Kümmerle J. M. Theiss F. Smith R. K. W. (2019). “Diagnosis and management of tendon and ligament disorders,” in Equine Surgery Philadelphia, WB, USA (WB Saunders), 1411–1445.

[B29] Liu X. Liu J. Zhao S. Zhang H. Cai W. Cai M. (2016). Interleukin-4 is essential for microglia/macrophage M2 polarization and long-term recovery after cerebral ischemia. Stroke 47 (2), 498–504. Epub 2016 Jan 5. PMID: 26732561; PMCID: PMC4729613. 10.1161/STROKEAHA.115.012079 26732561 PMC4729613

[B30] Loh E. Y. X. Mohamad N. Fauzi M. B. Ng M. H. Mohd Amin M. C. I. (2018). Development of a bacterial cellulose-based hydrogel cell carrier containing keratinocytes and fibroblasts for full-thickness wound healing. Sci. Rep. 8 (1), 2875. PMID: 29440678; PMCID: PMC5811544. 10.1038/s41598-018-21174-7 29440678 PMC5811544

[B31] Lovati A. B. Bottagisio M. Moretti M. (2016). Decellularized and engineered tendons as biological substitutes: a critical review. Stem cells Int. 2016, 1–24. 10.1155/2016/7276150 PMC4736572 26880985

[B32] Ma X. (2001). TNF-alpha and IL-12: a balancing act in macrophage functioning. Microbes Infect. 3 (2), 121–129. PMID: 11251298. 10.1016/s1286-4579(00)01359-9 11251298

[B33] Pluim M. Martens A. Vanderperren K. Sarrazin S. Koene M. Luciani A. (2018). Short- and long term follow-up of 150 sports horses diagnosed with tendinopathy or desmopathy by ultrasonographic examination and treated with high-power laser therapy. Res. Vet. Sci. 119, 232–238. 10.1016/j.rvsc.2018.06.003 30005398

[B34] Pongratz U. Licka T. (2017). Algometry to measure pain threshold in the horse's back - an *in vivo* and *in vitro* study. BMC Veterinary Res. 13 (1), 80. PMID: 28356118; PMCID: PMC5372265. 10.1186/s12917-017-1002-y PMC5372265 28356118

[B35] Ribitsch I. Baptista P. M. Lange-Consiglio A. Melotti L. Patruno M. Jenner F. (2020). Large animal models in regenerative medicine and tissue engineering: to do or not to do. Front. Bioeng. Biotechnol. 8, 972. PMID: 32903631; PMCID: PMC7438731. 10.3389/fbioe.2020.00972 32903631 PMC7438731

[B36] Ring E. F. J. Ammer K. Diakides N. A. Bronzino J. D. (2008). “Thermal imaging in the disease of the skeletal and neuromuscular systems,” in Medical infrared imaging Boca Raton, FL, USA (CRC Press).

[B37] Saldin L. T. Cramer M. C. Velankar S. S. White L. J. Badylak S. F. (2017). Extracellular matrix hydrogels from decellularized tissues: structure and function. Acta biomater. 49, 1–15. 10.1016/j.actbio.2016.11.068 27915024 PMC5253110

[B38] Santos V. H. Pfeifer J. P. H. de Souza J. B. Milani B. H. G. de Oliveira R. A. Assis M. G. (2018). Culture of mesenchymal stem cells derived from equine synovial membrane in alginate hydrogel microcapsules. BMC veterinary Res. 14 (1), 114. 10.1186/s12917-018-1425-0 PMC5870504 29587733

[B39] Sheikh Z. Brooks P. Barzilay O. Fine N. Glogauer M. (2015). Macrophages, foreign body giant cells and their response to implantable biomaterials. Materials 8, 5671–5701. 10.3390/ma8095269 28793529 PMC5512621

[B40] Shojaee A. Parham A. (2019). Strategies of tenogenic differentiation of equine stem cells for tendon repair: current status and challenges. Stem Cell Res. Ther. 10 (1), 181. 10.1186/s13287-019-1291-0 31215490 PMC6582602

[B41] Smith R. D. Carr A. Dakin S. G. Snelling S. J. Yapp C. Hakimi O. (2016). The response of tenocytes to commercial scaffolds used for rotator cuff repair. Eur Cell Mater 31, 107–18. 10.22203/ecm.v031a08 26815643

[B42] Spang M. T. Christman K. L. (2018). Extracellular matrix hydrogel therapies: *in vivo* applications and development. Acta biomater. 68, 1–14. 10.1016/j.actbio.2017.12.019 29274480 PMC5857190

[B43] Van Den Eeckhout B. Tavernier J. Gerlo S. (2021). Interleukin-1 as innate mediator of T cell immunity. Front. Immunol. 11, 621931. PMID: 33584721; PMCID: PMC7873566. 10.3389/fimmu.2020.621931 33584721 PMC7873566

[B44] Visser E. M. Menke E. S. Van Loon J. P. (2019). Pressure algometry for assessment of abdominal wall sensitivity in horses after ventral midline coeliotomy. Veterinary Anaesth. Analg. 46 (6), 820–828. Epub 2019 Jun 10. PMID: 31570274. 10.1016/j.vaa.2019.03.008 31570274

[B45] Visser J. Levett P. A. te Moller N. C. Besems J. Boere K. W. van Rijen M. H. (2015). Crosslinkable hydrogels derived from cartilage, meniscus, and tendon tissue. Tissue Eng. Part A 21 (7-8), 1195–1206. Epub 2015 Feb 9. PMID: 25557049; PMCID: PMC4394887. 10.1089/ten.TEA.2014.0362 25557049 PMC4394887

[B46] Witherel C. E. Abebayehu D. Barker T. H. Spiller K. L. (2019). Macrophage and fibroblast interactions in biomaterial-mediated fibrosis. Adv. Healthc. Mater. 8 (4), e1801451. Epub 2019 Jan 18. PMID: 30658015; PMCID: PMC6415913. 10.1002/adhm.201801451 30658015 PMC6415913

[B47] Yang G. Rothrauff B. B. Lin H. Gottardi R. Alexander P. G. Tuan R. S. (2013). Enhancement of tenogenic differentiation of human adipose stem cells by tendon-derived extracellular matrix. Biomaterials 34 (37), 9295–9306. 10.1016/j.biomaterials.2013.08.054 24044998 PMC3951997

[B48] Yin H. Caceres M. D. Yan Z. Schieker M. Nerlich M. Docheva D. (2019). Tenomodulin regulates matrix remodeling of mouse tendon stem/progenitor cells in an *ex vivo* collagen I gel model. Biochem. Biophysical Res. Commun. 512, 691–697. 10.1016/j.bbrc.2019.03.063 30922565

[B49] Zachary F. J. (2021). Pathologic basis of veterinary disease. 7th Edition. Amsterdam, Netherlands Elsevier.

[B50] Zhang G. Zhou X. Hu S. Jin Y. Qiu Z. (2022). Large animal models for the study of tendinopathy. Front. Cell Dev. Biol. 10, 1031638. PMID: 36393858; PMCID: PMC9640604. 10.3389/fcell.2022.1031638 36393858 PMC9640604

[B51] Zhang S. Ju W. Chen X. Zhao Y. Feng L. Yin Z. (2021). Hierarchical ultrastructure: an overview of what is known about tendons and future perspective for tendon engineering. Bioact. Mater 8, 124–139. PMID: 34541391; PMCID: PMC8424392. 10.1016/j.bioactmat.2021.06.007 34541391 PMC8424392

[B52] Zhu L. Zhao Q. Yang T. Ding W. Zhao Y. (2015). Cellular metabolism and macrophage functional polarization. Int. Rev. Immunol. 34 (1), 82–100. PMID: 25340307. 10.3109/08830185.2014.969421 25340307

