# Supplementary material for: Biocompatibility of hydrogel derived from equine tendon extracellular matrix in horses subcutaneous tissue
Source: Front Bioeng Biotechnol. 2024 Jan 8;11:1296743. doi: 10.3389/fbioe.2023.1296743 (PMC10801062; doi:10.3389/fbioe.2023.1296743)
Supplement: Supplementary file 1 [file Presentation1.PPTX]

## Slide 1
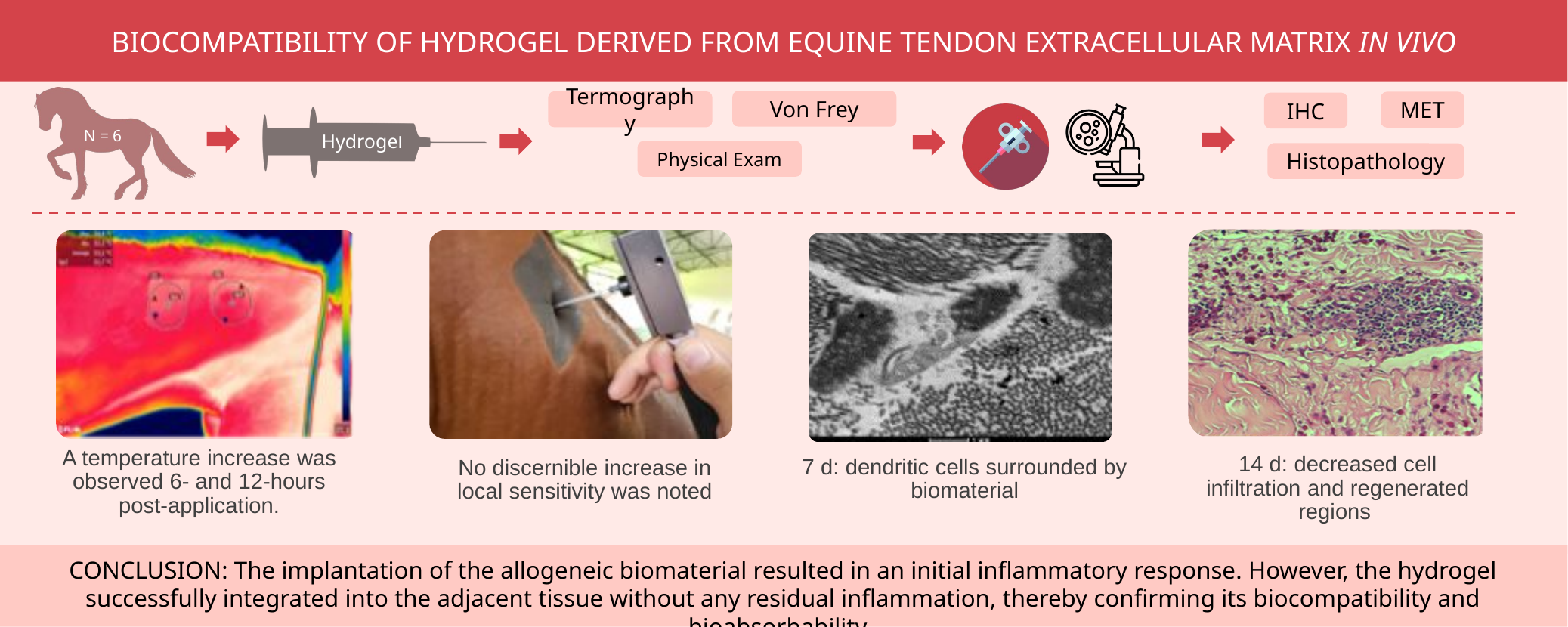

BIOCOMPATIBILITY OF HYDROGEL DERIVED FROM EQUINE TENDON EXTRACELLULAR MATRIX IN VIVO
Von Frey
Termography
MET
IHC
N = 6
Hydrogel
Physical Exam
Histopathology
A temperature increase was observed 6- and 12-hours post-application.
14 d: decreased cell infiltration and regenerated regions
No discernible increase in local sensitivity was noted
7 d: dendritic cells surrounded by biomaterial
CONCLUSION: The implantation of the allogeneic biomaterial resulted in an initial inflammatory response. However, the hydrogel successfully integrated into the adjacent tissue without any residual inflammation, thereby confirming its biocompatibility and bioabsorbability.
